# A Rare Case of an Intra-nasal Ectopic Tooth in a Young Woman

**DOI:** 10.7759/cureus.19370

**Published:** 2021-11-08

**Authors:** Rajamani Anand, Alexander Kieu, Einstein Arulraj, Gurubharath Ilangovan, Narmada D.A., Gaana AshwathNarayana, Meera A Alneyadi, Moien A.B. Khan

**Affiliations:** 1 Radiology, Chettinad Hospital and Research Institute, Chennai, IND; 2 Family Medicine, United Arab Emirates University, Al Ain, ARE; 3 Family Medicine, Kanad Hospital, Al Ain, ARE; 4 Radiology, Chettinad Academy of Research and Education, Chennai, IND; 5 Radiology, Chettinad Hospital and Research Institute Chettinad Academy of Research and Education, Chennai, IND; 6 Radiology, Chettinad Hospital and Research Institute, Chettinad Academy of Research and Education, Chennai, IND; 7 Family Medicine, College of Medicine and Health Sciences, United Arab Emirates University, Al Ain, ARE; 8 Primary Care, North West London - National Health Service, London, GBR

**Keywords:** hyposmia, nasal tooth, mesiodens, ectopic tooth, supernumerary tooth

## Abstract

Teeth in non-dentate areas including the intra-sinus and intranasal teeth are rarely encountered in clinical practice. Although the majority of patients remain asymptomatic, the usual presenting complaints are nasal obstruction, epistaxis, hyposmia and headache. In this article, we present a case of an intranasal tooth in a 15-year-old female who presented with complaints of hyposmia and nasal obstruction. Computed tomography (CT) of the paranasal sinuses and nasal cavity showed a tooth-like structure in the left inferior nasal cavity extending from the hard palate. The mainstay of treatment is the surgical removal of the ectopic tooth under anaesthesia. Even in asymptomatic patients, surgical removal of the nasal tooth is advised to prevent complications. Along with a clinician’s understanding of the condition, imaging aids in the diagnosis of an ectopic tooth. Imaging, particularly with CT, also helps plan the surgical approach to treatment.

## Introduction

The intranasal tooth is an uncommon encounter in clinical practice. Ectopic or supernumerary teeth can occur in 0.1%-1% of the population [1], but the incidence of ectopic teeth in the nasal cavity is especially rare [2,3]. Mesiodens is a supernumerary tooth present in the palatal midline at the site of the incisors. The predisposing factors for an ectopic nasal tooth are trauma, ectopic eruption, supernumerary teeth, odontogenic or rhinogenic infections, and they may occur in patients with cleft lip or palate [4,5]. The clinical manifestations of an intranasal tooth may vary. Patients can be asymptomatic and diagnosed on routine clinical and radiological examination. Symptomatic patients complain of hyposmia, nasal obstruction, chronic sinusitis, rhinorrhea, a nasal mass, headache and facial pain. Computed tomography (CT) scan with reconstruction plays the main role in the diagnosing and surgical planning of an intranasal tooth.

## Case presentation

A 15-year-old girl presented with chronic complaints of nasal obstruction and hyposmia on the left side. The patient had nasal obstruction symptoms for 6 months; before which she was asymptomatic. She was taking self-administered medications without relief. She had no history of maxillo-facial surgery or trauma in the past. She had no relevant family history or congenital anomalies. Upon clinical examination of the nose, there was a bump along the floor of the left nasal cavity, and her intraoral dentition appeared normal.

**Figure 1:**
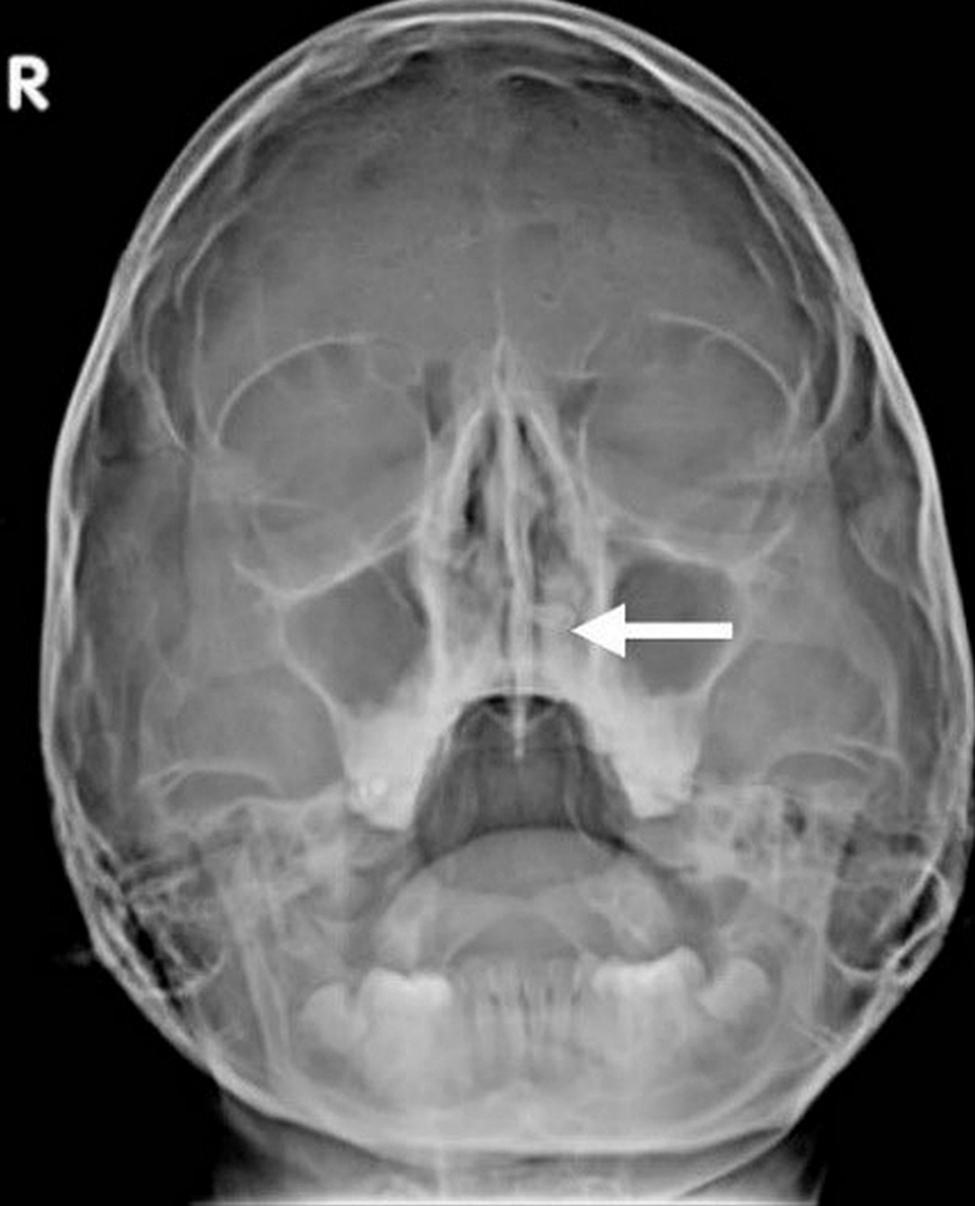
Plain x-ray shows the radiopaque structure in the left nasal cavity, embedded in the hard palate (white arrow).

A plain radiograph of the paranasal sinus and nasal cavity was performed which showed a radiopaque focus in the left nasal cavity (Figure 1).

**Figure 2:**
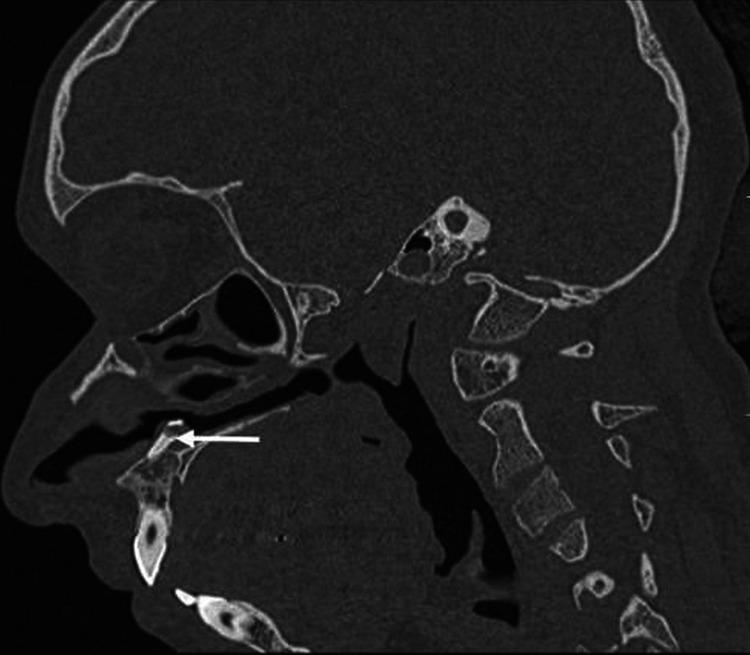
Sagittal image showing complete extension of an intranasal tooth (white arrow), which is embedded in the hard palate.

**Figure 3:**
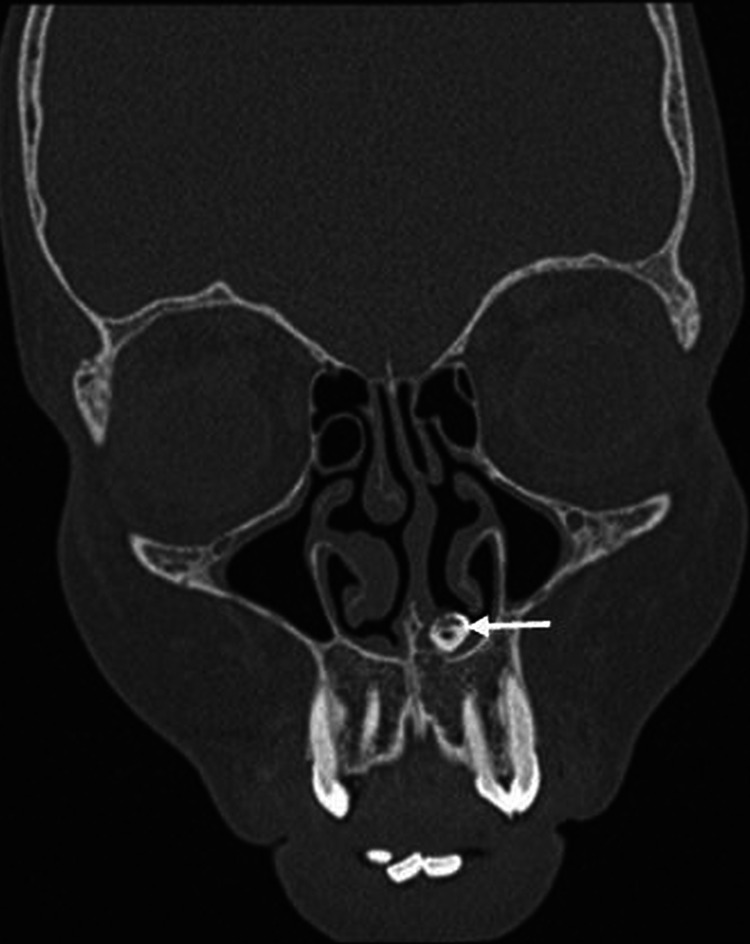
Coronal image showing a bone-like structure (white arrow) embedded in the hard palate extending into the left nasal cavity.

**Figure 4:**
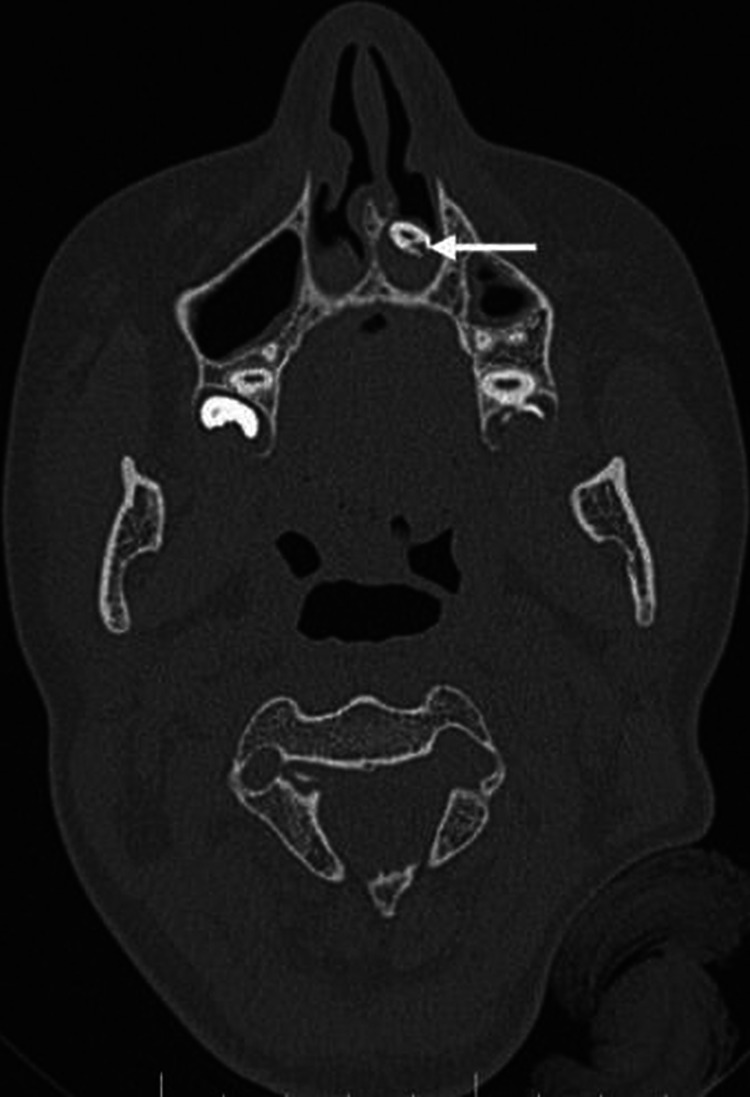
Axial CT scan showing an ectopic tooth in the left nasal cavity surrounded by soft tissue (white arrow).

The CT scan of paranasal sinuses showed a tooth-like bony structure with a pulp cavity in the hard palate extending into the left inferior nasal cavity and a deviated nasal septum with convexity to the left (Figures 2-4); a shape resembling a canine with a relatively smaller size.

 

 

**Figure 5:**
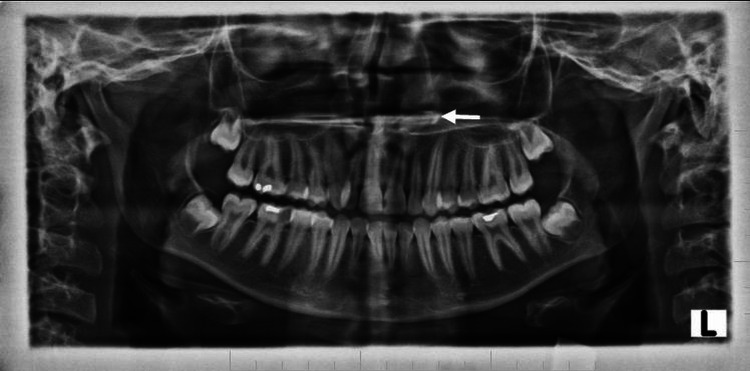
Orthopantomogram showing tooth-like radiopaque structure (white arrow); a shape resembling a canine with a relatively smaller size. The radiodensity of this radiopaque structure resembles enamel, dentin, the pulp chamber and the pulp canal in relation to the left nasal floor.

**Figure 6:**
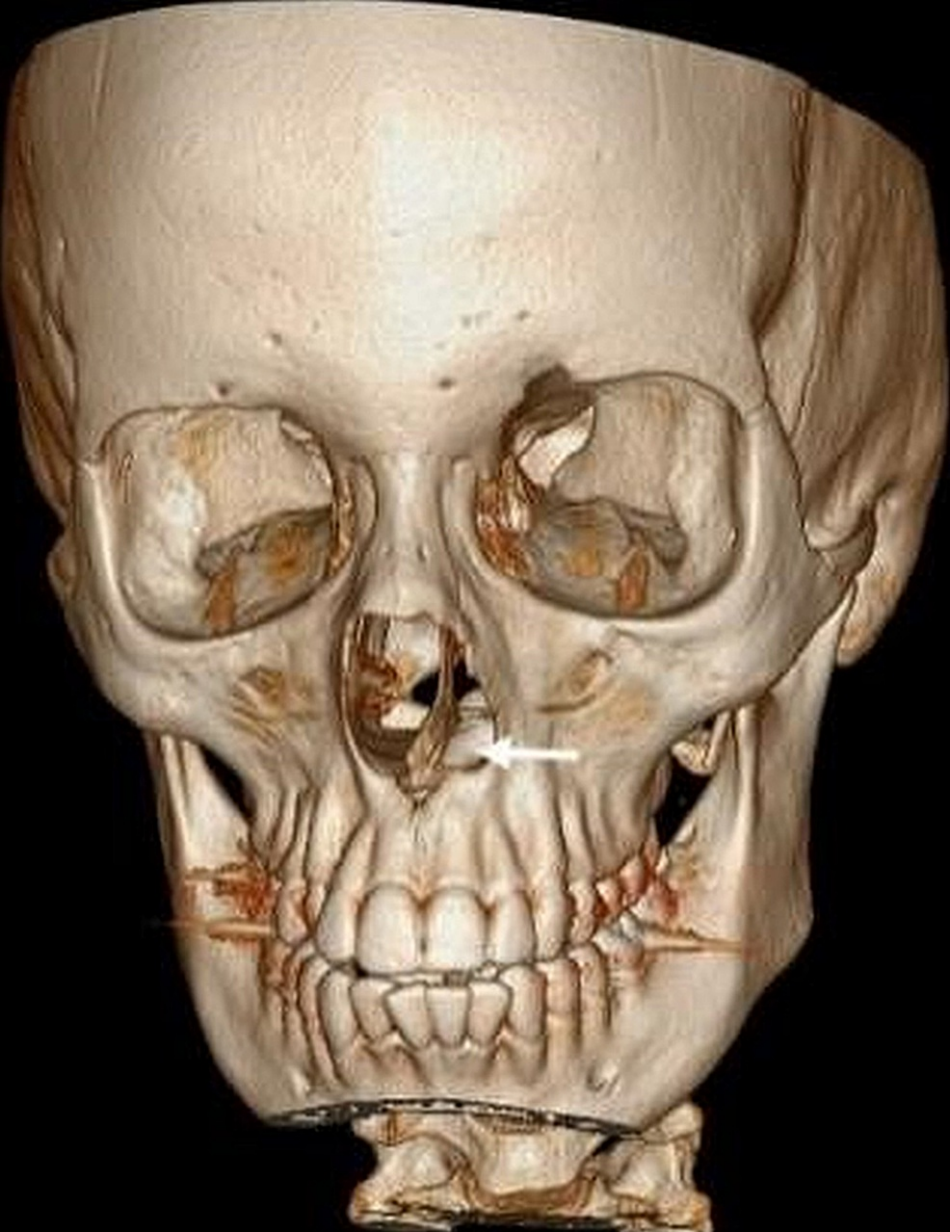
Reconstructed 3D CT image showing tooth-like structure in the left nasal cavity (white arrow) embedded in the hard palate. 3D CT - Three-dimensional computed tomography scan

Orthopantomogram showed a tooth-like radiopaque structure (white arrow) (Figure 5). A three-dimensional computed tomography (3D CT) scan showed a tooth-like structure in the left nasal cavity (Figure 6).

This intranasal tooth was supernumerary. Mild mucosal thickening was noted in the bilateral maxillary and sphenoid sinuses. No tooth-like structures were found on the right side. All other teeth appeared normal. The patient had complete resolution of nasal obstruction and hyposmia following endoscopic removal of the ectopic intranasal tooth.

## Discussion

Ectopic teeth presenting outside the oral cavity can be deciduous, permanent or supernumerary teeth. The incidence of ectopic or supernumerary teeth accounts for 0.1-1%, a rare clinical entity [1,2]. The most commonly affected sites are the maxillary sinus and the palate, whereas the nasal cavity, coronoid process, mandibular condyle and orbits are rarely affected. When ectopic teeth are located in the upper incisor area, it is known as mesiodens, which has the appearance of a malformed peg-like tooth. The upper incisor region is the most commonly affected site [4].

A large number of anterior supernumerary teeth remain unerupted. The ectopic teeth can appear in an inverted form, and they are heterotopic teeth whose edges are reverted towards the alveolar ridge.

There are several theories for the development of ectopic teeth. One theory is the theory of developmental origin. Primates have three pairs of incisors. The theory suggests that the development of ectopic tooth is due to the reversion of dentine similar to extinct primates. Another theory is the dental lamina hyperactivity theory which states that the supernumerary teeth grew from the third tooth bed. According to the dental lamina hyperactivity theory, an intranasal tooth can emerge from the dental lamina near the permanent tooth bud or the splitting of the permanent tooth bud [1,3].

Another theory is that supernumerary tooth growth represents a reversion to the three-incisor dentition of extinct primates [1,3]. The presence of an ectopic tooth has been linked to blockage during tooth eruption due to crowded dentition, persisting deciduous teeth or thick bone.

The aetiology of the intranasal tooth is broadly classified into two groups according to the problems in tooth germ development and migration. The predisposing factors for ectopic nasal teeth are trauma, supernumerary teeth, odontogenic or rhinogenic infections, cleft lip or cleft palate [2]. Ectopic teeth can grow and appear as additional teeth on the palate, or they might grow into the nasal cavity.

Extra teeth have a horizontal or vertical atypical crown. This condition may be asymptomatic or may cause various symptoms like hyposmia, nasal obstruction, facial pain, epistaxis and headache. Ectopic teeth can lead to multiple complications like naso-oral fistula, rhinitis caseosa with perforation, external nasal deformities and fungal infections such as aspergillosis. The clinical and radiological examination helps in diagnosing intranasal teeth. Intranasal teeth might clinically present as a white mass surrounded by granulation tissue or as an external bump along the floor of the nasal cavity on the affected side, as seen in our case [6].

The CT findings of an intranasal tooth in our case appeared as a radiodense structure embedded in the hard palate, seen extending into the left nasal cavity. The above-mentioned radio dense structure showed the same attenuation as that of oral teeth. This ectopic tooth is presented with a pulp cavity seen as a hypodense area in the centre, which is seen better in bone window settings and surrounded by soft tissue indicating granulation tissue.

The other clinical conditions mimicking intranasal teeth include foreign body, rhinolith, infections such as tuberculosis and aspergillosis with calcification, nasal polyp with calcification, benign tumours such as osteoma, hemangioma and malignant tumours such as osteosarcoma and chondrosarcoma [7-9]. Although intranasal ectopic teeth are uncommon, they can cause complications if left untreated.

CT scanning is used to diagnose and plan treatment. Surgical or endoscopic removal of an ectopic tooth is recommended as a treatment [10]. An intranasal tooth may rarely present with a bony socket in the floor of the nose which is difficult to extract surgically. In this situation, the depth of the eruption is evaluated with the help of CT imaging, and the intranasal tooth is removed once the root of the permanent teeth is entirely formed to minimize injury and complications during development.

## Conclusions

A computed tomography scan is helpful in evaluating the depth of eruption and for planning treatment for an intranasal tooth. Surgical removal or endoscopic extraction under microscopic control of intranasal tooth is advised to prevent symptoms and complications. If the tooth is in the nasal cavity, minor surgery is necessary; however, if the tooth is in a bony socket on the floor of the nose, extraction may be difficult. Intranasal teeth should be surgically extracted if patients are symptomatic after the roots of permanent teeth have fully developed to avoid damage and complications during development. A combined clinical and radiological approach helps to accurately diagnose the condition and plan the appropriate management.
